# Genome Sequence of *Erysipelothrix* sp. Strain Poltava, Isolated from Acute Septic Erysipelas of Swine in Ukraine

**DOI:** 10.1128/mra.00438-22

**Published:** 2022-08-02

**Authors:** Oleksandr Tarasov, Ganna Kovalenko, Larysa Muzykina, Maksym Bezymennyi, Eric Bortz, Devin M. Drown

**Affiliations:** a National Academy of Agrarian Sciences of Ukraine, Institute for Veterinary Medicine (IVM), Kyiv, Ukraine; b Department of Biological Sciences, University of Alaska Anchorage, Anchorage, Alaska, USA; c Division of Virology, Department of Pathology, University of Cambridge, Cambridge, United Kingdom; d Department of Biology and Wildlife, University of Alaska Fairbanks, Fairbanks, Alaska, USA; e Institute of Arctic Biology, University of Alaska Fairbanks, Fairbanks, Alaska, USA; Loyola University Chicago

## Abstract

The complete genome of *Erysipelothrix* sp. strain Poltava, isolated from fatal acute septic erysipelas of swine in Ukraine, was assembled using Nanopore sequences. One circular chromosome of 1,794,858 bp (*N*_50_, 1,794,858 bp) encodes 16 putative antibiotic resistance genes and secreted virulence factors, highlighting the risk of cross-species livestock and human infection.

## ANNOUNCEMENT

As part of a scientific initiative to understand differential swine infections that cocirculate with African swine fever, in Ukraine (1), we sequenced the genome of an *Erysipelothrix* sp. strain isolated in 2006 from domestic swine with an acute septicemic form of swine erysipelas from a backyard farm in Ukraine (Poltava Oblast, Shyshaky Raion; 49°52′40.5948″N, 34°3′51.5262″E) (2). The sequencing of this strain using an Oxford Nanopore Technologies (ONT) MinION platform in veterinary laboratories in Ukraine represents a genomic exploration of a collection of archived bacterial isolates, providing historical and contemporary insight into circulating livestock pathogens (3–5).

This isolate was collected from tissue samples from swine mesenteric lymphatic nodes and spleen in 2006 and cultured in brain heart infusion (BHI) agar (M1611; HiMedia) and in selective medium, modified blood-azide medium (CM0259; Oxoid). The culture was incubated under aerobic conditions at 37°C for 24 h. Biochemical tests were performed using the API Coryne test (BioMeriex, France) (6), and PCR was performed as a confirmatory test using the primers ER1 and ER2 (7), identifying this isolate as Erysipelothrix rhusiopathiae (class *Erysipelotrichia*, phylum *Firmicutes*), a Gram-positive rod bacterium that often presents as erysipelas and in severe cases causes acute septicemia, or chronic endocarditis with polyarthritis, leading to severe wasting disease in swine (8). The stock culture was stored lyophilized at −20°C, subcultured, and relyophilized every 24 months. After growth for 24 h in BHI agar, a colony was resuspended in 200 μL PBS, incubated for 1.5 h at 37°C, followed by washing, and resuspended in demineralized water. Three sample vials from the same isolate were pooled under biosafety level 2 (BSL2) for DNA isolation, using a DNeasy UltraClean microbial kit (Qiagen).

We used 495 ng of DNA as input for a rapid sequencing library (SQK-RAD004; ONT) and sequenced it on an R9.4.1 flow cell (FLO-MIN106; flow cell ID FAL31485) for 16 h using a MinION Mk1B device. We base called the raw data using Guppy v6.1.3 (ONT) in super-accuracy mode (-c dna_r9.4.1_450bps_sup.cfg), filtering reads with a quality below 10 (–min_qscore 10) for an output of 1,080,139,073 bp in 1,047,659 reads with a read length *N*_50_ value of 2,654 bp. When accounting for only reads passing the quality filter, the run generated 770,677,927 bp in 524,171 reads with a read length *N*_50_ value of 3,130 bp and a median Q score of 12.4. We used Filtlong v0.2.0 (https://github.com/rrwick/Filtlong) to subset 50% of the reads (-keep_percent 50) and prioritize them by read quality (–mean_q_weight 10). After filtering, we had 385,339,219 bp in 132,040 reads with a read length *N*_50_ value of 4,664 bp and a median Q score of 14.5.

We assembled the genome *de novo* using Flye v2.9 (9) with the 385-Mb quality-controlled data set (coverage, 213×), specifying high-quality Nanopore reads (–nano-hq). Our initial assembly contained one contig, identified as circular using Flye. We corrected the assembly using Medaka v1.6.0 (https://github.com/nanoporetech/medaka), specifying base-caller model (-m r941_min_sup_g507). Our 1,794,858-bp (*N*_50_, 1,794,858 bp) polished assembly consists of a single circular contig (GC content, 36.39%). We rotated the circular genome start position to *dnaA* using Circlator v1.5.5 (10). Default parameters were used for all software unless otherwise specified.

During the data submission pipeline, the genome deposited at GenBank was annotated using PGAP v6.1 (11–13) and contained 53 tRNAs, 12 rRNAs, and 2,436 coding DNA sequences (CDS). CheckM (14) reported 86.94% completeness with 0.96% contamination. PATRIC v3.6.12 (15, 16) identified 16 antibiotic resistance genes (Table 1). Using the Comprehensive Genome Analysis service in PATRIC, a phylogenetic analysis found the isolate to be similar to members of the genus *Erysipelothrix*. We used FastANI (17) to calculate an average nucleotide identity of 98.86% to the most similar genome, Erysipelothrix rhusiopathiae strain Fujisawa (GenBank accession no. NC_015601) (8). We extracted a consensus sequence of the four full-length 16S rRNA gene copies to use as a query with blastn (18) against the NCBI 16S rRNA database. We found 99% identity to both Erysipelothrix piscisicarius strain 15TAL0474 (NR_170394) and Erysipelothrix rhusiopathiae strain ATCC 19414 (NR_040837). Therefore, we designated this isolate *Erysipelothrix* sp. strain Poltava.

**TABLE 1:** Antimicrobial resistance genes

AMR mechanism	Gene(s)
Antibiotic target in susceptible species	Alr, Ddl, EF-G, EF-Tu, folA, Dfr, gyrA, gyrB, Iso-tRNA, MurA, rpoB, rpoC, S10p, S12p
Gene conferring resistance via absence	gidB
Protein altering cell wall charge, conferring antibiotic resistance	PgsA

### Data availability.

This whole-genome sequencing project has been deposited at GenBank under the accession no. CP096542.1. The raw data for this project can be found under SRA accession no. SRR18770851 and BioProject accession no. PRJNA827134.
